# Hump‐Shaped Relationship Between Microbial Carbon Use‐Efficiency and Soil Organic Carbon in Alpine Grasslands

**DOI:** 10.1002/advs.202513917

**Published:** 2025-11-25

**Authors:** Yuting Wang, Yongneng Wei, Gangsheng Wang, Yang Ruan, Ling Li, Xiang Liu, Yunfeng Yang, Qirong Shen, Ning Ling

**Affiliations:** ^1^ State Key Laboratory of Herbage Improvement and Grassland Agro‐Ecosystems Centre for Grassland Microbiome Lanzhou University Lanzhou Gansu 730020 China; ^2^ Jiangsu Provincial Key Lab for Organic Solid Waste Utilization Jiangsu Collaborative Innovation Center for Solid Organic Waste Resource Utilization Nanjing Agricultural University Nanjing 210095 China; ^3^ Institute for Water‐Carbon Cycles and Carbon Neutrality Wuhan University Wuhan 430072 China; ^4^ Institute of Environment and Ecology Tsinghua Shenzhen International Graduate School Tsinghua University Shenzhen 518055 China

**Keywords:** ^18^O water‐labeling, large‐scale sampling, Qinghai–Tibetan Plateau, soil microbial carbon use efficiency, soil organic carbon pools

## Abstract

Microbial carbon use efficiency (CUE) mediates soil organic carbon (SOC) turnover, yet its drivers and role in carbon stabilization in alpine grasslands remain unclear. Using data from 45 sites across the Qinghai‐Tibetan Plateau and ^18^O‐water to analyze substrate‐independent CUE, this study reveals a hump‐shaped relationship between microbial CUE and SOC. Below an SOC threshold of 54 g C kg^−1^, increases in precipitation, plant biomass, soil total nitrogen, and phosphorus contents raise CUE, supporting microbial in vivo turnover. High CUE implies more carbon incorporation into microbial cells, facilitating mineral‐associated organic carbon (MAOC) formation via sorption on clays. This process is reflected by rising MAOC/SOC ratios with increasing clay content. Above the SOC threshold of 54 g C kg^−1^, phosphorus limitation and high clay content suppress CUE, with the influence of clay amplifying under wetter conditions. In carbon‐rich soils, the newly discovered inverse correlation between MAOC and microbial efficiency in alpine systems highlights that CUE only reflects the potential for carbon sequestration. These findings emphasize the need to balance soil carbon accumulation and nutrient availability for microorganisms to maintain the soil carbon storage capacity of climate‐sensitive ecosystems.

## Graphical abstract

On the Qinghai–Tibetan Plateau, microbial carbon use efficiency (CUE) peaks at intermediate soil organic carbon levels and declines thereafter. In carbon‐rich soils, the formation of stable mineral‐associated organic carbon is decoupled from microbial CUE. These unexpected relationships challenge existing models and highlight that optimizing soil carbon sequestration depends on maintaining microbial nutrient availability.

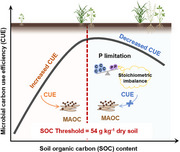

## 1 Introduction

Soil microorganisms, as pivotal agents in the global carbon cycle, exert dual regulatory roles in the soil organic carbon (SOC) cycle via two contrasting pathways: biomass synthesis that will be stabilized after microbial death (entombing effect, EE) vs enzymatic decomposition that accelerates organic carbon loss (decomposing effect, DE).^[^ ^1^ ^]^ Among microbial traits, carbon use efficiency (CUE)—defined as the ratio of carbon assimilated into biomass to the total carbon uptake^[^ ^2^, ^3^ ^]^—represents a crucial variable reflecting the balance between the first step toward EE and DE pathways. Critically, microbial CUE is not fixed but is responsive to external environmental drivers. Temperature, moisture, soil properties, and stoichiometry are important factors affecting CUE.^[^ ^4^, ^5^ ^]^ Among them, macroclimatic factors influence microbial activity and the cycle of carbon and nutrients. Substrate stoichiometry, particularly the balance between carbon and nutrients, influences microbial metabolic activities such as extracellular enzyme secretion,^[^ ^6^ ^]^ growth, and respiration.^[^ ^7^, ^8^ ^]^ When substrates are stoichiometrically balanced, microorganisms can efficiently convert assimilated carbon into biomass, leading to high CUE.^[^ ^9^, ^10^ ^]^ Conversely, stoichiometric imbalance (e.g., high SOC/total phosphorus (C/P) or SOC/total nitrogen (C/N) ratios) forces microorganisms to increase energy acquisition, leading to respiratory losses and a decline in CUE.^[^ ^11^, ^12^ ^]^ These imply that the dominant factors affecting CUE reorganize along substrate quality and environmental filters, ultimately determining the magnitude and direction of CUE changes.

Current investigations on microbial CUE primarily focus on soils under forest, croplands, and low‐altitude grassland ecosystems,^[^ ^5^, ^13^, ^14^ ^]^ leaving alpine ecosystems largely understudied. The Qinghai‐Tibetan Plateau, in particular, is a climate‐sensitive region dominated by ≈70% grasslands^[^ ^15^ ^]^ that store significant yet vulnerable soil carbon. The region's unique cold, low‐oxygen environment, coupled with frequent freeze–thaw cycles, tends to favor microbial strategies oriented toward survival.^[^ ^16^ ^]^ Critically, cold‐induced organic carbon accumulation magnifies the carbon quality effect.^[^ ^17^ ^]^ This is because low temperatures, slow carbon decomposition, and nutrient mineralization lead to the accumulation of carbon‐rich but nutrient‐deficient organic matter.^[^ ^18^, ^19^ ^]^ Studies in systems like subtropical croplands have shown opposite changes of CUE in low‐ and high‐carbon soils,^[^ ^11^ ^]^ hinting at potential nonlinearities. Whether such a nonlinear relationship exists in alpine ecosystems remains unknown. A systematic examination of CUE across an SOC gradient, as well as the mechanisms driving the SOC‐CUE relationship, is therefore essential for understanding the carbon cycling in these vulnerable ecosystems.

Recently, the ecological significance of CUE has remained contentious. Traditionally, high CUE was thought to raise mineral‐associated organic carbon (MAOC), a stable carbon pool associated with minerals, largely derived from microbial metabolites and necromass, and attributed to the entombing effect.^[^ ^1^, ^20^ ^]^ However, accumulating evidence suggests a more nuanced reality.^[^ ^21^, ^22^ ^]^ CUE facilitates short‐term carbon trapping in microbial biomass,^[^ ^23^ ^]^ whereas the capacity of soils to store MAOC is limited.^[^ ^24^ ^]^ As the accumulation rate of MAOC declines or even reaches zero, more carbon enters the particulate organic carbon (POC) pool—readily accessible to microorganisms—generally supporting a high CUE.^[^ ^25^ ^]^ This high CUE suppresses MAOC formation by promoting DE‐dominated negative feedbacks.^[^ ^25^ ^]^ Resolving this contradiction requires a clearer quantification of the linkage between CUE and the dynamics of the POC and MAOC pools across soil environments.

**Figure 1:**
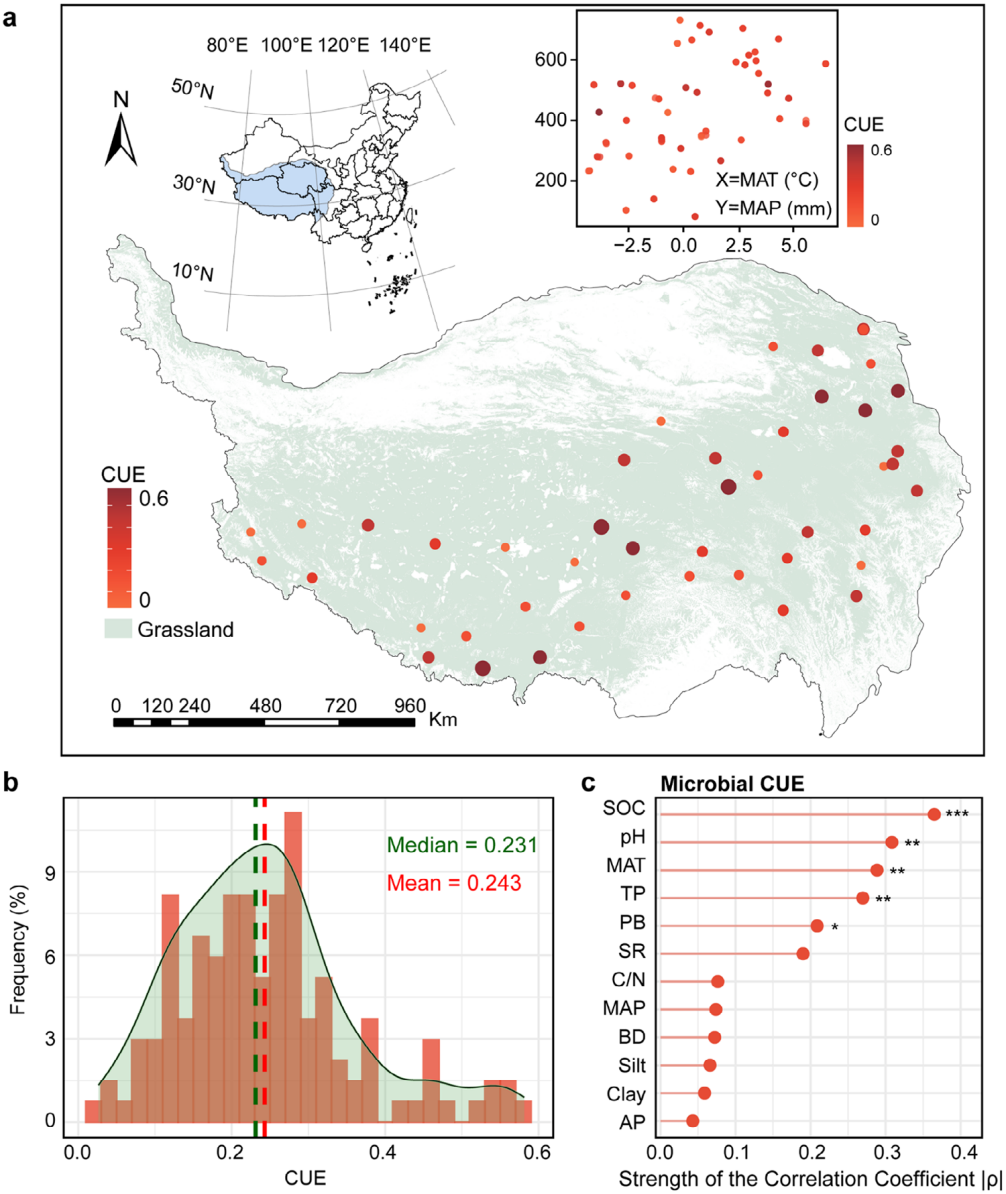
Geographic distribution of sampling sites and the relationship between soil microbial carbon use efficiency (CUE) and environmental variables. a) Map of 45 grassland sampling sites on the Qinghai‐Tibetan Plateau, with CUE values indicated by point size and color intensity. b) Frequency and range distributions of microbial CUE in 135 soils (45 sites × 3 plots). c) Partial correlation analysis. All variables were Z‐scored, and factors with strong collinearity (VIF >10) were removed (TN, C/P, and AI). The horizontal axis represents the strength of Spearman's correlation coefficient (|ρ|). Significance testing was performed using the Benjamini–Hochberg method for FDR‐adjusted; ^*^ *p* < 0.05; ^**^ *p* < 0.01; ^***^ *p* < 0.001. SOC, soil organic carbon; TN, soil total nitrogen; TP, soil total phosphorus; AP, soil available phosphorus; C/N, soil organic carbon to total nitrogen; C/P, soil organic carbon to total phosphorus; MAP, mean annual precipitation; MAT, mean annual temperature; AI, aridity index; BD, bulk density; PB, plant biomass; SR, plant richness.

Based on this framework, our study focused on the alpine grassland ecosystems of the Qinghai‐Tibetan Plateau to identify the multidimensional drivers of microbial CUE across an organic carbon content gradient and to assess how CUE is related to the SOC pools—especially MAOC. A sampling transect network, spanning 2230 km east–west and 1034 km north–south, was established across 45 sites, yielding 135 composite soil samples (45 sites × 3 subplots) and covering the majority of the plateau's grasslands (**Figure** **1**a). We quantified microbial CUE using ^18^O‐water tracers and simultaneously measured POC and MAOC. Potential drivers included climates (for example, mean annual temperature and precipitation), soil physico‐chemical properties (pH and bulk density), nutrient status (SOC, C/N ratio, C/P ratio, etc.), soil texture (silt and clay content), and plants (biomass and diversity). A combination of partial correlation analysis, model fitting (linear or generalized additive models), and piecewise structural equation modeling was used to analyze the pathways underlying the impact of environmental factors on CUE. Stratified linear model fitting was conducted to elucidate the associations between CUE and soil carbon pools. We hypothesized that the relationship between microbial CUE and SOC is nonlinear with a threshold, driven by distinct environmental factors that undergo reorganization across levels of soil carbon contents. This study advances our understanding of microbial carbon metabolism in alpine ecosystems, providing a theoretical basis for assessing the Qinghai‐Tibetan Plateau's carbon sink under global change.

## 2 Results

### 2.1 Distribution and Environmental Correlates of Microbial CUE in Alpine Grasslands

**Figure 2:**
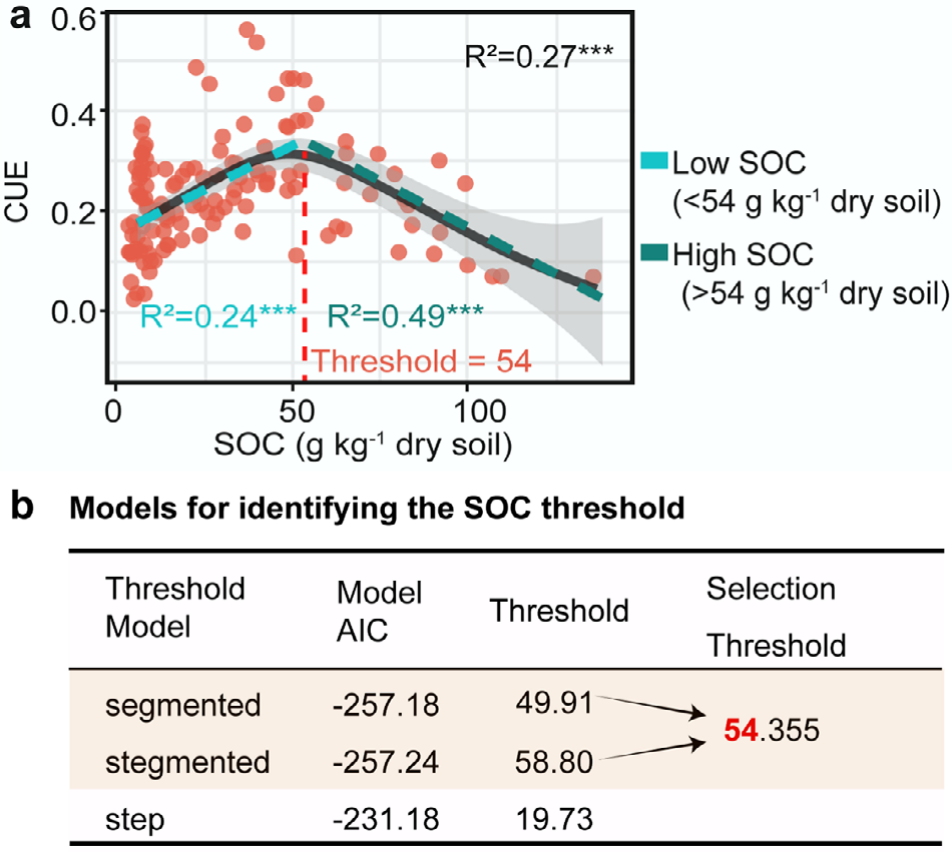
Relationship between soil organic carbon (SOC) and microbial carbon use efficiency (CUE). a) Hump‐shaped relationship. b) Models for identifying the SOC threshold. The nonlinear trends are fitted by a generalized additive model (GAM), while straight lines represent linear fits, with shaded areas showing confidence intervals. Red dashed lines and numbers indicate the identified SOC thresholds, and blue–green dashed lines show linear fits on either side of the threshold. ^*^ *p* < 0.05; ^**^ *p* < 0.01; ^***^ *p* < 0.001. Based on the Mahalanobis distance, three outliers of SOC were excluded. The SOC threshold was identified by comparing the Akaike Information Criterion (AIC) values of three threshold regression models (“step,” “stegmented,” and “segmented”). Lower AIC values indicate a better fit of the model. A difference in AIC values of less than 2 between the two models suggests no substantial difference; therefore, the final threshold for SOC was determined as the mean of the thresholds from both models.

Microbial CUE in 135 soils (45 sites × 3 plots) from the grasslands of the Qinghai‐Tibetan Plateau ranged from 0.03 to 0.58, with an average of 0.24 (Figure 1a,b; Table S1, Supporting Information). In 90% of the sites, CUE values did not exceed 0.40 (Figure 1b). The partial correlation, controlling for all other variables, showed that SOC, pH, mean annual temperature (MAT), plant biomass (PB), and soil total phosphorus content (TP) were correlated with CUE (Figure 1c). In contrast, first‐order partial correlations (controlling each covariate individually) showed that only SOC retained a correlated with CUE (Figure S1, Supporting Information). Notably, a distinct “hump‐shaped” relationship emerged between SOC and CUE (R^2^ = 0.27, *p* < 0.001; **Figure** **2**a). As SOC increased, CUE initially rose (R^2^ = 0.24, *p* < 0.001) and then declined (R^2^ = 0.49, *p* < 0.001), with a threshold at 54 g C kg^−1^ (Figure 2b; Table S2, Supporting Information).

### 2.2 Changes in Abiotic and Biotic Factors on Either Side of the SOC Threshold

Based on the SOC threshold, all soils were classified into low SOC (SOC < 54 g C kg^−1^) and high SOC (SOC >54 g C kg^−1^) categories. Mean annual precipitation (MAP), aridity index, soil silt and clay contents, total carbon (TC), total nitrogen (TN), total phosphorus (TP), available phosphorus (AP), C/N and C/P ratios, plant biomass, and plant richness were all higher in high SOC soils (*p* < 0.05), whereas soil pH, bulk density, and sand content were lower (*p* < 0.05; Figure S2, Supporting Information). Plant biomass, and plant richness, soil TN, and TP increased with the rising SOC gradient (3.8–142 g C kg^−1^) (Table S1 and Figure S3, Supporting Information).

### 2.3 Drivers of the Nonlinear Relationship Between SOC and Microbial CUE

**Figure 3:**
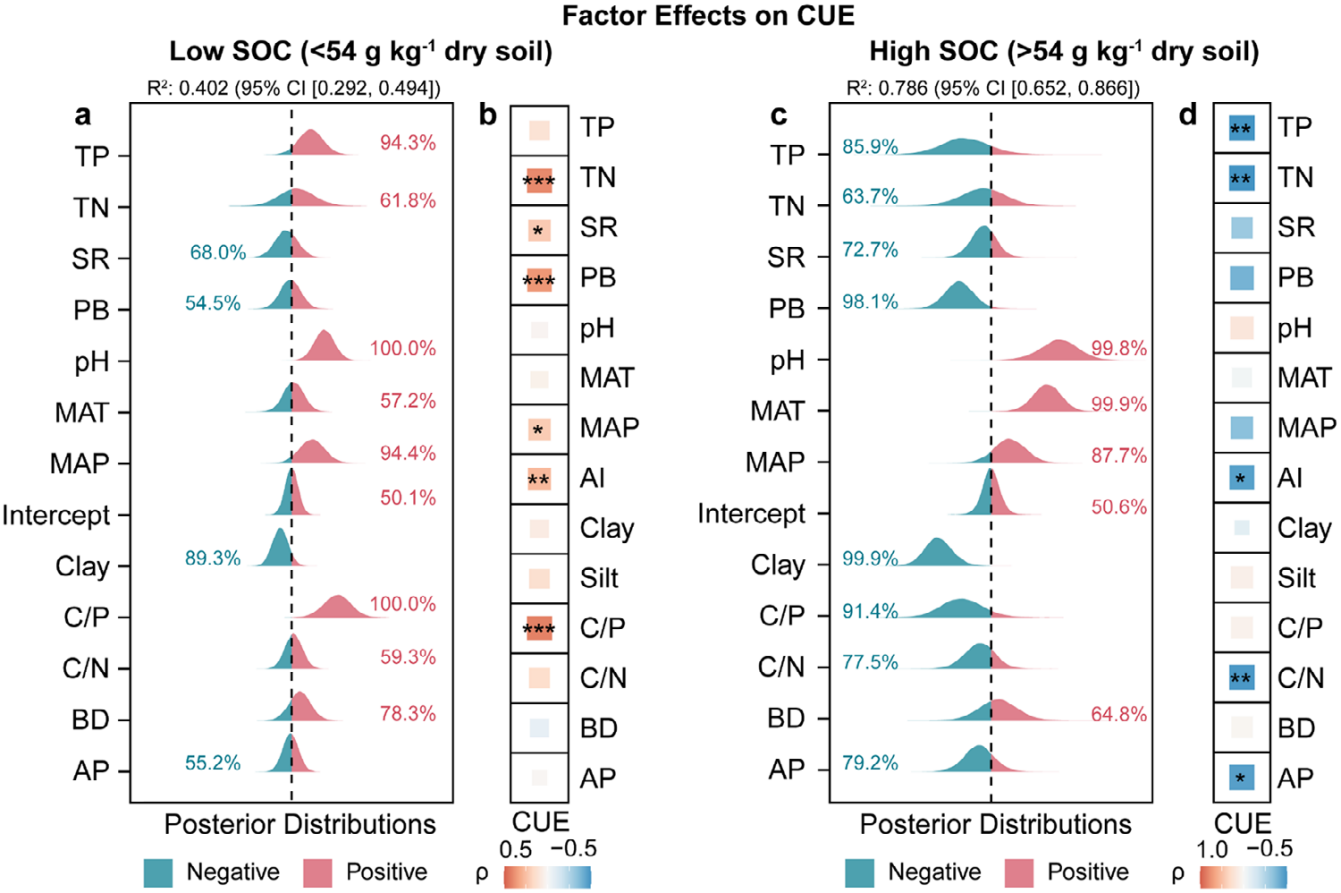
The influence of environmental factors on microbial carbon use efficiency (CUE) in a,b) low and c,d) high organic carbon soils. (a,c) Bayesian posterior distribution plot: variables with VIF >10 (AI and silt) are excluded. The half‐eye plot shows the posterior distribution of the variable, with negative effects shown in blue and positive effects in red. The value represents the posterior probability (Pr) of impact on CUE, and Pr >90% is considered meaningful. The explanatory power of the Bayesian model is represented by Bayesian R^2^, which better quantifies the uncertainty in model explanation. The figure shows the median of the Bayesian R^2^ along with its 95% credible interval (CI). (b,d) The heatmaps depict the Spearman correlations between microbial CUE and the environmental factors. ^*^ *p* < 0.05; ^**^ *p* < 0.01; ^***^ *p* < 0.001. TN, soil total nitrogen; TP, soil total phosphorus; AP, soil available phosphorus; C/N, soil organic carbon to total nitrogen; C/P, soil organic carbon to total phosphorus; MAP, mean annual precipitation; MAT, mean annual temperature; AI, aridity index; BD, bulk density; PB, plant biomass; SR, plant richness.

To investigate the distinct drivers of microbial CUE between low and high SOC soils, a multi‐model analysis was conducted, excluding SOC content from the explanatory variables. In low SOC soils, the Bayesian model results showed that soil TP, pH, C/P ratio, and MAP increased CUE (posterior probability >90%; **Figure** **3**a), with a Bayesian R^2^ of 0.402 (95% credible interval [0.292, 0.494]). CUE also increased with soil TN, plant biomass, and plant richness (*p* < 0.05; Figure 3b). In high SOC soils, plant biomass, soil clay content, and the C/P ratio decreased CUE (posterior probability >90%), while pH and MAT increased it (posterior probability >90%; Figure 3c), with a Bayesian R^2^ of 0.786 (95% credible interval [0.652, 0.866]). The linear fitting relationship between soil C/P ratio and CUE was weak (R^2^ = 0.03, *p* >0.05; Figure S4, Supporting Information), but soil C/P ratio was an effective factor of CUE in Bayesian models (Figure 3c). Moreover, CUE declined with soil TP, TN, AP, C/N ratio, and aridity index (*p* < 0.05; Figure 3d).

**Figure 4:**
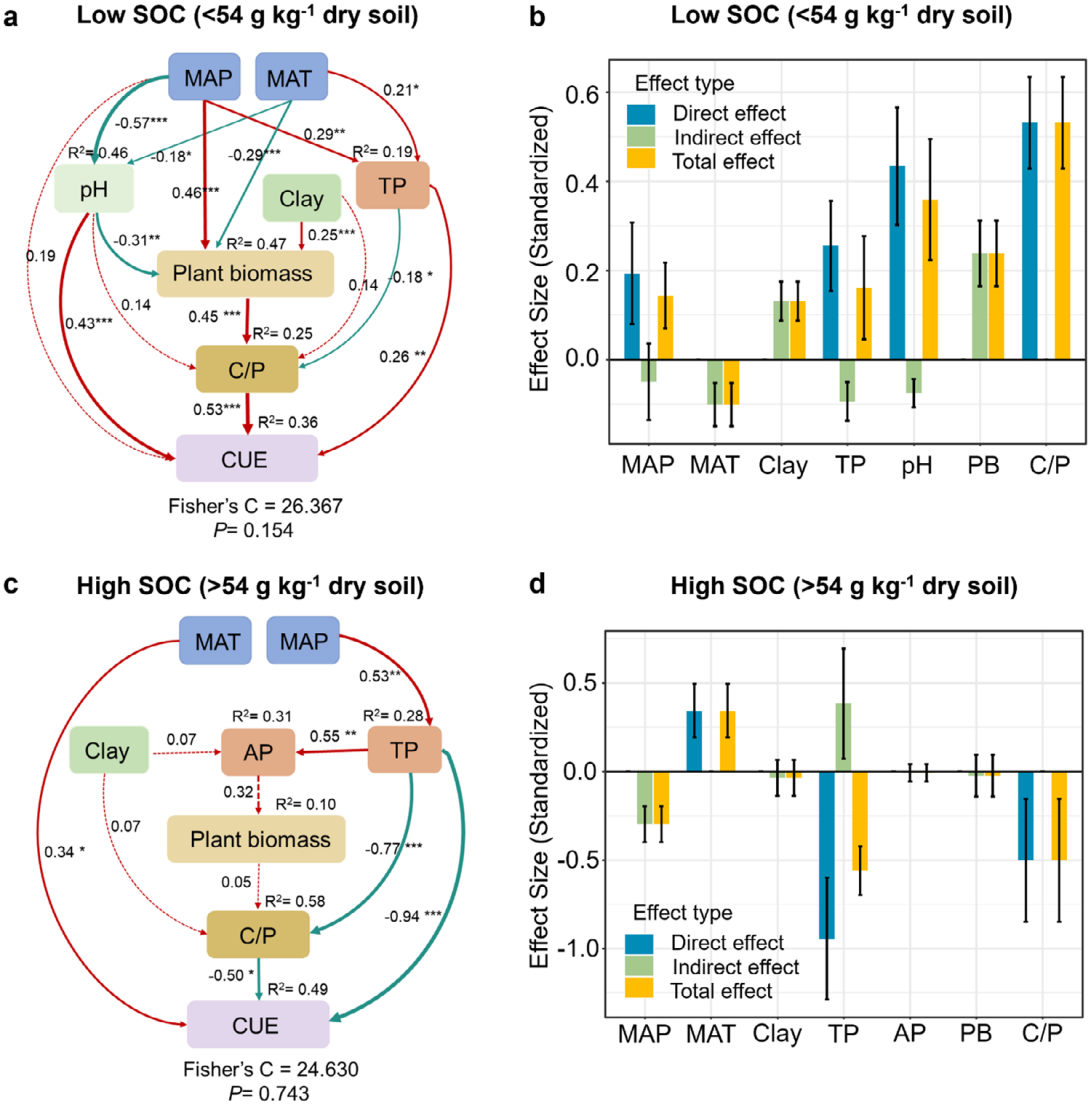
Direct and indirect effects of explanatory variables on microbial carbon use efficiency (CUE) in a,b) low and c,d) high organic carbon soils. The path diagrams (a,c) include standardized coefficients and R^2^. The thickness of the arrows indicates the strength of the effect, and the color represents the direction of influence, with red indicating positive effects and green indicating negative effects. The Fisher's C value assesses model quality. ^*^ *p* < 0.05; ^**^ *p* < 0.01; ^***^ *p* < 0.001. The standardized effect diagrams (b,d) display the total, direct, and indirect effects of each variable. Error bars denote bootstrap standard errors (B = 5000). MAP, mean annual precipitation; MAT, mean annual temperature; TP, soil total phosphorus; AP, soil available phosphorus; C/P, soil organic carbon to total phosphorus; PB, plant biomass.

The piecewise structural equation model was constructed to assess the potential direct and indirect effects of explanatory variables on microbial CUE (**Figure** **4**). In low SOC soils, MAP increased plant biomass, with a path coefficient of 0.46 (*p* < 0.05; Figure 4a), and plant biomass enhanced CUE indirectly by increasing the soil C/P ratio (*p* < 0.05; Figure 4a). The indirect effect of plant biomass on CUE was also influenced by clay content. Both pH and TP directly increased CUE, with path coefficients of 0.43 and 0.26, respectively (*p* < 0.05; Figure 4a). The total effects of the soil C/P ratio and plant biomass on CUE were 0.53 and 0.24, respectively (Figure 4b; Table S3, Supporting Information).

In soils with high organic carbon content, MAT directly increased CUE, with a path coefficient of 0.34 (*p* < 0.05; Figure 4c). However, TP and the soil C/P ratio reduced CUE, with path coefficients of −0.94 and −0.50, respectively (*p* < 0.05; Figure 4c). The indirect effect of soil clay content on CUE through C/P was weak (path coefficient: 0.07, Figure 4c). The aridity index intensified the effect of clay on CUE reduction (Figure S5, Supporting Information), defined as the ratio of precipitation to potential evapotranspiration. A higher aridity index indicates wetter conditions. The total effects of TP and the soil C/P ratio on CUE were −0.56 and −0.50, respectively (Figure 4d; Table S4, Supporting Information).

### 2.4 Linking Changes in Soil Organic Carbon Pools to Microbial CUE

**Figure 5:**
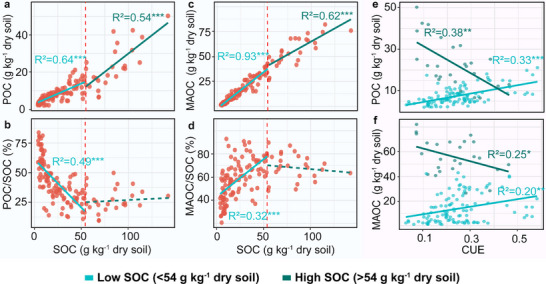
Relationships among soil carbon fractions, organic carbon content, and microbial carbon use efficiency (CUE). a) Particulate organic carbon (POC) and soil organic carbon (SOC), b) POC/SOC and SOC, c) mineral‐associated organic carbon (MAOC) and SOC, d) MAOC/SOC and SOC, e) POC and CUE, and f) MAOC and CUE. Solid lines indicate statistically supported fits; green dashed lines denote nonsignificant fits; the red dashed line marks the SOC threshold at 54 g C kg^−1^ dry soil; ^*^ *p* < 0.05; ^**^ *p* < 0.01; ^***^ *p* < 0.001.

POC, MAOC, and the MAOC/SOC ratio increased along the SOC gradient (**Figure** **5**a,c,d), while the POC/SOC ratio declined in low SOC soils (Figure 5b). In this context, both POC and MAOC increased with CUE (Figure 5e,f). MAOC and MAOC/SOC increased with increasing clay content in low SOC soils (Figure S6, Supporting Information). In contrast, in high SOC soils, both POC and MAOC also increased with SOC (Figure 5a,c); however, the proportions of POC/SOC and MAOC/SOC remained stable, with POC/SOC being ≈25% and MAOC/SOC being 70% consistently (Figure 5b,d). MAOC and clay content had no relationship (Figure S6, Supporting Information). Under these conditions, POC and MAOC decreased with CUE (Figure 5e,f).

## 3 Discussion

Microbial CUE across the Qinghai‐Tibetan Plateau alpine grasslands ranged from 0.03 to 0.58 (Figure 1a,b; Table S1, Supporting Information). In cold‐climate soils, microbes with low CUE reflect a “survival over growth” strategy. Microbial strategies, however, are influenced not only by climate but also by soil conditions. SOC had the strongest correlation with microbial CUE (Figure 1c). SOC content should not be interpreted merely as a quantitative carbon pool; rather, it serves as an integrative indicator that reflects climatic conditions—which regulate plant productivity—and soil properties, including texture, structure, and nutrient stoichiometry.^[^ ^26^, ^27^ ^]^ Consequently, here, SOC is treated as a state variable, analogous to temperature or elevation. The spatial gradient in SOC content (3.8–142 g C kg^−1^; Table S1, Supporting Information) corresponds to distinct shifts in climatic, edaphic, and plant properties (Figure S2, Supporting Information), establishing a natural heterogeneity suited for investigating microbial metabolic efficiency.

### 3.1 Mechanistic Interpretation of Nonlinear Relationship Between Microbial CUE and SOC Content

A key finding is the identification of a “hump‐shaped” relationship between microbial CUE and SOC through empirical correlative analysis (Figure 2a), which is interpreted as a phenomenological trend rather than causal. This “hump‐shaped” relationship captures a previously under‐documented decline in CUE beyond a specific SOC threshold (54 g C kg^−1^ dry soil; Figure 2b). This supported our hypothesis that the relationship between microbial CUE and SOC is nonlinear with a threshold, initially driven by nutrients and plant biomass input, and transitioning to phosphorus (P) limitation and high clay content. Our data resonate with recent theoretical advances,^[^ ^25^ ^]^ which challenge the simplistic view of a linear linkage between microbial CUE and SOC.

Based on the SOC threshold, all samples were divided into high and low organic carbon soils, which differ in climate, soil properties, and plant characteristics (Figure S2, Supporting Information). In the analysis of the drivers for CUE variation, SOC and its fractions were excluded to avoid causal overlap. Microbial CUE declined with excess carbon resources (Figure 2a). This counterintuitive response revealed ecological constraints associated with resource oversupply. P limitation from carbon excess reduced microbial CUE in high‐SOC soils (Figures 3c and 4c,d). Although a simple bivariate correlation between soil C/P ratio and CUE was not statistically significant (Figure S4, Supporting Information), the major control of C/P on CUE in Bayesian models (Figure 3c) highlights the advantage of Bayesian methods for handling noisy ecological data with limited sample sizes compared with frequentist approaches.^[^ ^28^, ^29^ ^]^ Microbial activity and growth depend on the stoichiometrically balanced supply of key elements in the environment, notably carbon (C), nitrogen (N), and P.^[^ ^30^ ^]^ The substrate C/P ratio exceeds the elemental threshold required for microbial metabolism, triggering P limitation and diverting C surplus toward overflow respiration, thereby reducing CUE.^[^ ^4^, ^31^ ^]^ Although some non‐steady‐state microbial populations can adjust their cellular stoichiometry to buffer nutrient imbalances, this capacity is constrained by physiological limits.^[^ ^32^ ^]^ In alpine ecosystems, such regulatory flexibility appears particularly restricted,^[^ ^33^ ^]^ rendering CUE more responsive to shifts in nutrient limitation.

Clay content was another factor that decreased microbial CUE in soils with high organic C content (Figure 3c). The effect of clay on CUE was not mediated by exacerbating P limitation (Figure 4c), but rather was amplified by the aridity index (Figure S5, Supporting Information), defined as MAP/potential evapotranspiration (PET). Mechanistically, high‐clay soils readily form microscale diffusion constraints and localized anaerobic microsites,^[^ ^34^, ^35^ ^]^ particularly under high soil moisture, thereby increasing microbial metabolic costs and reducing CUE. Organic C‐rich soils had high clay and silt contents alongside humid conditions, as indicated by high MAP and aridity index (Figure S2, Supporting Information).

In low organic C soils, microbial CUE increased with SOC (Figure 2a). For heterotrophic microbial communities, organic carbon or energy is typically the primary limiting factor for growth.^[^ ^36^ ^]^ According to Blackman's Limitation Theory, biological growth is constrained by the most stronger limiting resource.^[^ ^37^ ^]^ Under conditions of extremely low SOC and plant biomass, microbial metabolism face energy starvation. Limited energy sources are allocated solely to maintenance rather than to microbial growth,^[^ ^38^ ^]^ resulting in a low CUE. Plant biomass increased along the SOC content, gradually alleviating energy limitations, accompanied by an increase in total P and N contents (Figure S3, Supporting Information), which had a clear stimulatory influence on CUE (Figure 3a,b). Previously, synergistic increases in resource have been shown to raise microbial CUE.^[^ ^39^ ^]^ MAP increased plant biomass, contributing to a higher CUE (Figure 4a). In low organic C soils, the effect of plant biomass increase on the C/P ratio reflects a balance between biomass‐driven organic matter inputs and the soil's nutrient supply. This is indicated by the increase in CUE with TP and by the fact that soil C/P ratios are lower than those in high SOC soils (Figure S2n, Supporting Information). Additionally, through root exudates such as organic acids, sugars, and enzymes, plants provide nutrients and dissolve minerals,^[^ ^40^ ^]^ thereby supplying readily accessible resources for microbes.

Together, these findings indicated a mechanistic shift in alpine ecosystems: from a “plant C and nutrient synergistic increase” under moderate SOC content to a dual mechanism of “P limitation and diffusion constraints under high clay content” at high SOC content, forming the observed hump‐shaped SOC–CUE relationship. From a microbial life‐history strategies perspective, this nonlinear relationship might reflect a strategic shift from a high growth yield strategy (Y‐strategy, prioritizing growth efficiency and biomass production) to a resource acquisition strategy (A‐strategy, prioritizing enzyme‐mediated resource acquisition) under changing substrate and nutrient constraints.^[^ ^41^ ^]^

### 3.2 Linking Environmentally Driven CUE with Soil Carbon Stability: Insights and Prospects

Microbial CUE is a central parameter mediating the fate and stabilization of SOC.^[^ ^42^ ^]^ Higher CUE is generally associated with increased anabolism and reduced respiratory loss,^[^ ^43^ ^]^ favoring greater C allocation to microbial biomass and later to necromass, which are major contributors to MAOC^[^ ^23^ ^]^ through their role in forming organo‐mineral complexes.^[^ ^44^ ^]^ Within this theoretical framework, MAOC increased synchronously with microbial CUE in low SOC soils (Figure 5f). The observation that MAOC represented an increasing fraction of SOC in low C soils (Figure 5d) is explained by the rise in clay content (Figure S6, Supporting Information), which provides the essential mineral surfaces for MAOC formation.^[^ ^24^ ^]^

Notably, our findings revealed a novel decoupling between microbial CUE and MAOC under high C‐rich soils, a phenomenon previously observed under certain global change drivers^[^ ^45^ ^]^ but not yet characterized in alpine ecosystems. The decline in MAOC with increasing CUE (Figure 5f) suggested a shift in dominant carbon allocation pathways from microbial assimilation to abiotic preservation in high SOC soils. This finding challenged the assumption that CUE is a consistent predictor of stable carbon formation and underscored that CUE reflects only the potential for carbon retention.^[^ ^46^, ^47^ ^]^ The MAOC formation in this context relies more heavily on preservation mechanisms, such as mineral sorption and physical occlusion, rather than immediate microbial transformation,^[^ ^23^ ^]^ leading to MAOC formation that is less dependent on in vivo microbial turnover.^[^ ^24^ ^]^ MAOC has nothing to do with clay content in high SOC soils (Figure S6, Supporting Information), indicating limited availability of mineral adsorption sites. The ongoing accumulation of MAOC (Figure 5c,d) supports the previously reported mechanism of organic matter multilayer stacking.^[^ ^48^ ^]^ In this stacking pathway, the increase in MAOC relies more on the physicochemical properties of existing organic matter. This interpretation is further supported by recent work,^[^ ^22^ ^]^ which demonstrated that MAOC accumulation is primarily promoted by existing MAOC content and saturation deficit, rather than by microbial CUE. These insights emphasize the need to integrate both microbial efficiency and abiotic stabilization processes when assessing soil carbon dynamics under changing environmental conditions.

Collectively, this study provides empirical evidence of a hump‐shaped relationship between microbial CUE and SOC across the Qinghai‐Tibetan Plateau. Microbial CUE was initially increased by the rise in plant C and nutrients. Above the SOC threshold, however, CUE was suppressed due to both P limitation and high clay content. Our results support a model in which microbial CUE can promote SOC accumulation up to a threshold, beyond which C stabilization becomes decoupled from microbial efficiency. These findings emphasize the need for a mechanistic understanding of the complex relationship between soil carbon pools and microbial metabolism under environmental regulation. They also highlight the importance of balancing soil carbon accumulation with microbial nutrient availability in the design of ecosystem management and carbon sequestration strategies, especially in sensitive alpine regions undergoing rapid environmental change.

## 4 Experimental Section

### Study Area and Sample Collection

This study involved large‐scale sampling of grasslands across the Qinghai‐Tibet Plateau, spanning an east‐west range (28.32 to 37.64 °N, 79.82 to 102.60 °E; Figure 1a). The survey covered 45 field sites, with elevations ranging from 2505 to 4961 m (a gradient of 2456 m). The mean annual temperature (MAT) at the sites spanned from −4.02 to 6.69 °C, while mean annual precipitation (MAP) ranged from 81 to 733 mm (Table S1, Supporting Information). The aridity index (AI), defined as the ratio of precipitation to potential evapotranspiration, was obtained from the Global‐AI_PET_v3 database (https://doi.org/10.6084/m9.figshare.7504448.v5). Higher AI values indicate wetter conditions, while lower values indicate drier environments.^[^ ^49^ ^]^ Soil properties, such as bulk density (BD) and silt and clay contents, were obtained from the SoilGrids web portal (https://soilgrids.org/). It was important to note that SoilGrids‐derived data offer estimates at a ≈250 m resolution, which may not adequately capture fine‐scale variability at the individual plot level. However, these data were appropriate for examining trends at the regional scale.

During the months of July and August in 2022 and 2023, soil samples from locations far from urban areas were collected that can represent natural vegetation. 45 sampling sites were selected based on the following criteria: i) the sites were at least 50 km apart; and ii) each site was a minimum of 500 m away from major roads. At each site, three 0.5 m × 0.5 m subplots were randomly established at three of the four corners of a 50 m × 50 m square. Geographic coordinates (latitude, longitude, and elevation) were recorded at each subplot. From each subplot, six soil cores (0–15 cm depth) were collected using a 38‐mm diameter auger and mixed thoroughly to form one composite sample. In total, 135 composite samples (45 sites × 3 subplots) were collected and transported to the laboratory in ice‐cooled containers.

Upon arrival at the laboratory, stones and plant roots were removed, and the soil was sieved through a 2‐mm mesh. The samples were then divided into two portions. One portion was air‐dried to measure total carbon, organic carbon, total nitrogen (TN), total phosphorus (TP), and available phosphorus (AP) contents; pH; particulate organic carbon (POC); and mineral‐associated organic carbon (MAOC). The other portion was stored at 4 °C for soil microbial carbon use efficiency (CUE) assessment.

### Analysis of Microbial CUE in Soils

Soil microcosms were constructed to perform both ^18^O‐labeled and conventional water incubation experiments. To ensure standardized conditions, fresh soil samples were pre‐incubated in the dark at 15 °C with moisture adjusted at 45% of water‐holding capacity (WHC) for one week. Following pre‐incubation, two 500 mg subsamples of each soil were transferred into sterile 2‐mL Biosharp microcentrifuge tubes. The ^18^O‐water (97 at%), where “at%” denotes atom percent (meaning that 97% of the oxygen atoms are ^18^O), was added to one aliquot to achieve a final ^18^O concentration of 20 at% and to adjust the soil moisture to 60% of WHC. The other aliquot received an equal volume of unlabeled sterile deionized water to serve as a natural abundance control. The tubes were placed open in 50‐mL incubation bottles and then immediately sealed. A blank control (without soil) was included for every three samples. All samples were incubated in the dark at 15 °C for 24 h.

After incubation, 25 mL of gas was extracted from each bottle with a syringe and stored in gas bags for analysis. The carbon dioxide (CO_2_) concentration was immediately determined using a gas chromatograph (Agilent 7890A GC, Agilent Technologies, Santa Clara, USA). The soil samples in the Biosharp tubes were snap‐frozen in liquid nitrogen and stored at −80 °C for downstream DNA extraction. Total DNA was extracted from the soil using the FastDNA Spin Kit for Soil (MP Biomedicals) following detailed protocols described in previous studies.^[^ ^50^, ^51^ ^]^ The DNA concentration was quantified with a Qubit 4.0 fluorometer. For isotopic analysis, the DNA extract was transferred to silver capsules, dried at 60 °C overnight, and folded. Total oxygen content and ^18^O abundance in the DNA were measured with a high‐temperature pyrolysis‐IRMS system (TC/EA‐IRMS, Thermo Fisher, USA).

Soil microbial biomass carbon (MBC) was quantified using the chloroform fumigation‐extraction method.^[^ ^52^ ^]^ Fumigated and non‐fumigated soils were extracted using 0.5 m K_2_SO_4_ (200 rpm, 30 min) after a 24‐h fumigation, with a soil‐to‐water ratio of 1:4. The filtered extracts were analyzed for organic carbon content using a total organic carbon analyzer (Elementar, Thermo Fisher Vario TOC). MBC was expressed as the difference between organic carbon contents in fumigated and non‐fumigated samples divided by 0.45, which served as the conversion factor.^[^ ^53^ ^]^

Soil CUE was determined using a substrate‐independent method based on tracing the incorporation of ^18^O from water into microbial DNA.^[^ ^54^ ^]^ This approach allowed the estimation of microbial growth rates.^[^ ^55^ ^]^ The soil microbial growth rate (C_growth_, µg C g^−1^ soil h^−1^) was calculated by dividing the amount of MBC produced during the incubation period. Previous studies had shown that a linear relationship exists between MBC and microbial DNA production, defined as the fDNA ratio.^[^ ^56^ ^]^ The amount of DNA produced during the incubation period was calculated based on the abundance of ^18^O in i) the labeled DNA, ii) the non‐labeled DNA (natural abundance), and iii) the soil water of the labeled sample. This DNA production was then converted to MBC using the fDNA ratio, which allowed the estimation of microbial growth rates.^[^ ^51^ ^]^ The soil microbial respiration rate (C_respiration_, µg C g^−1^ soil h^−1^) was determined from the amount of CO_2_ generated over the 24‐h incubation.^[^ ^51^ ^]^ Under the steady‐state assumption, soil CUE was calculated as CUE = C_growth_ / (C_growth_ + C_respiration_).^[^ ^57^ ^]^

### Determination of Soil Properties

Soil moisture content was determined by drying fresh soil at 105 °C to a constant weight. Soil pH was determined using an FE28‐Standard pH meter (Mettler Toledo, Shanghai, China) in CO_2_‐free water with a soil‐to‐water ratio of 2.5:1. Total carbon and nitrogen contents in the soil were analyzed using a Flash Smart elemental analyzer (Thermo Fisher, Waltham, MA, USA). AP content was analyzed using an automated discrete chemical analyzer (Smartchem 450) following a previously described method.^[^ ^58^ ^]^ TP content was determined using the automated discrete chemical analyzer after digesting the sample with sulfuric acid and perchloric acid. SOC was quantified via dry combustion using an elemental analyzer (Skalar, Breda, Netherlands).

POC and MAOC isolation was done using wet‐sieving and size fractionation methods.^[^ ^59^ ^]^ Briefly, air‐dried soil (6 g) was dispersed in 30 mL sodium hexametaphosphate solution (5 g L^−1^) with sufficient glass beads and subjected to an 18‐h oscillation for aggregate dissociation. The resulting suspension was rinsed through a 53‐µm mesh to separate mineral‐associated organic matter (MAOM, < 53 µm) from particulate organic matter (POM, >53 µm). The fractions were dried at 60 °C prior to mass determination. After grinding and sieving, the samples were subjected to carbonate elimination via acid fumigation,^[^ ^60^ ^]^ and organic carbon was quantified in both fractions using the elemental analyzer.

### Statistical Analysis

Spearman's partial correlation analysis was used to quantify the independent relationships between factors and microbial CUE. All variables were standardized using the Z‐score. Multicollinearity was assessed using the variance inflation factor (VIF), with SOC retained throughout the process. Variables were iteratively removed until all retained variables had a VIF below 10. The “*ppcor*” package in R was then used to calculate the full‐ and first‐order partial correlation coefficients (ρ) between each factor and CUE, controlling for the influence of other variables. Correlation strength was reported as the absolute value of the coefficient (|ρ|). Significance testing was performed using the Benjamini–Hochberg method for false discovery rate (FDR) correction.

The factors identified by the partial correlation analysis were selected as being associated with microbial CUE and conducted individual regression analyses to explore the relationships between these factors and CUE. Linear and nonlinear (quadratic and generalized additive model, GAM) models were fitted between each factor and CUE, selecting the optimal model based on the Akaike Information Criterion (AIC). Generally, a difference in AIC values greater than 2 indicated a significant difference between models, with the model with the lowest AIC considered the best fit (Table S5, Supporting Information). The GAM was implemented using the “*mgcv*” package in R. Model performance was evaluated based on both explanatory power (R^2^) and the trend of the fitted curves. Given the clear relationship observed between CUE and SOC (R^2^ = 0.27, *p* < 0.001), which has reliable explanatory and ecological significance, the main figure of this study highlights this fit. Details of the fits for other variables are provided in Figure S7 (Supporting Information).

Then, the threshold for the nonlinear relationship between SOC and CUE was attempted to be identified using the “*segmented*” and “*chngpt*” packages in R. Three threshold models, “step,” “stegmented,” and “segmented,” were tested, selected the best model based on AIC, and determined its corresponding threshold (Table S2, Supporting Information). Lower AIC values indicate a better fit of the model. A difference in AIC values of less than 2 between the two models suggests no substantial difference. Linear fitting was performed on the data on both sides of the threshold to simplify complex relationships.

After determining the SOC threshold, the Mann‐Whitney *U* test was used to analyze the differences in climatic, soil, and plant indicators on either side of the threshold. Based on this threshold, all samples were categorized into low and high‐organic‐carbon soils. To identify the primary factors driving changes in microbial CUE under low and high SOC soils, Bayesian regression modeling and Spearman correlation analysis were used to examine the effects of various explanatory variables on CUE, excluding SOC content from the analysis. The Bayesian model was built using the “*brms*” package in R.^[^ ^61^ ^]^ To ensure comparability and numerical stability, all variables were standardized (Z‐scores), and predictor variables with a variance inflation factor (VIF) greater than 10 were excluded. Markov chain Monte Carlo (MCMC) methods were used for sampling the posterior distribution, with four independent chains of 10000 iterations each. The first 5000 iterations were discarded as a warm‐up, and the remaining 5000 iterations were used for analysis. The adapt_delta parameter was set to 0.99 for better sampling efficiency. Convergence was checked by examining trace plots and ensuring the convergence statistic (R‐hat) was below 1.01. To assess the certainty of the effect direction, the posterior probability (Pr) was calculated using the “*dplyr*” package in R. The Bayesian R^2^ was calculated using the r2_bayes() function from the R package “*performance*.” This metric was estimated based on the model's posterior distribution and reflects the Bayesian model's ability to explain the variability of the response variable.^[^ ^62^ ^]^ Spearman correlation analysis was performed using the cor function from the “*stats*” package in R. This method is ideal for exploring monotonic trends between variables without assuming a specific data distribution. To ensure comparability and remove scale effects, all continuous variables within each subset were standardized.

Based on the Bayesian model and Spearman correlation analysis selecting variables, a piecewise structural equation model^[^ ^63^ ^]^ was constructed to evaluate the direct and indirect effects of environmental factors on CUE using the “*piecewiseSEM*” package in R. All data were standardized before analysis. An initial prior model was developed and refined by adding or removing paths until the model fit met chi‐squared test criteria (*p* >0.05). The direct, indirect, and total effects of predictor variables on response variables were calculated using Wright's path tracing rules. The standard error (SE) was quantified using a non‐parametric bootstrap approach with 5000 iterations. All statistical analyses were performed using R 4.4.1.

## Conflict of Interest

The authors declare no conflict of interest.

## Author Contributions

N.L. was responsible for the conception of the project. N.L. and Y.R. conceived of the methodology. X.L., Y.N.W., L.L., and Y.T.W. performed the investigation. Y.T.W. was responsible for visualization. Q.R.S. supervised the project. Y.T.W. and N.L. wrote the original draft of the manuscript. G.S.W., Y.F.Y., X.L., and N.L. contributed to reviewing and editing.

## Supporting information



Supporting Information

Supporting Information

## Data Availability

The data that support the findings of this study are available in the supplementary material of this article.
