# Supplementary material for: Hump‐Shaped Relationship Between Microbial Carbon Use‐Efficiency and Soil Organic Carbon in Alpine Grasslands
Source: Adv Sci (Weinh). 2025 Nov 25;13(8):e13917. doi: 10.1002/advs.202513917 (PMC12884716; doi:10.1002/advs.202513917)
Supplement: Supplementary file 1 — Supporting Information [file ADVS-13-e13917-s001.docx]

Supporting Information

**Hump-Shaped Relationship between Microbial Carbon Use-Efficiency and Soil Organic Carbon in Alpine Grasslands**

*Yuting Wang ^1^, Yongneng Wei ^2^, Gangsheng Wang ^1, 3^, Yang Ruan ^2^, Ling Li ^2^, Xiang Liu ^1^, Yunfeng Yang ^4^, Qirong Shen ^2^, Ning Ling ^1, 2, *^*

**Affiliations:**

^1^ *State Key Laboratory of Herbage Improvement and Grassland Agro-Ecosystems, Centre for Grassland Microbiome, Lanzhou University, Lanzhou, 730020, Gansu, China.*

^2^ *Jiangsu Provincial Key Lab for Organic Solid Waste Utilization, Jiangsu Collaborative Innovation Center for Solid Organic Waste Resource Utilization, Nanjing Agricultural University, Nanjing, 210095, China.*

^3^ *Institute for Water-Carbon Cycles and Carbon Neutrality, Wuhan University, Wuhan 430072, China.*

^4^ *Institute of Environment and Ecology, Tsinghua Shenzhen International Graduate School, Tsinghua University, Shenzhen 518055, China.*

*** Corresponding authors:**

Ning Ling, *E-mail address: nling@njau.edu.cn*

**This file includes:**

Supplementary Figures 1 to 7

Supplementary Tables 1 to 5

**
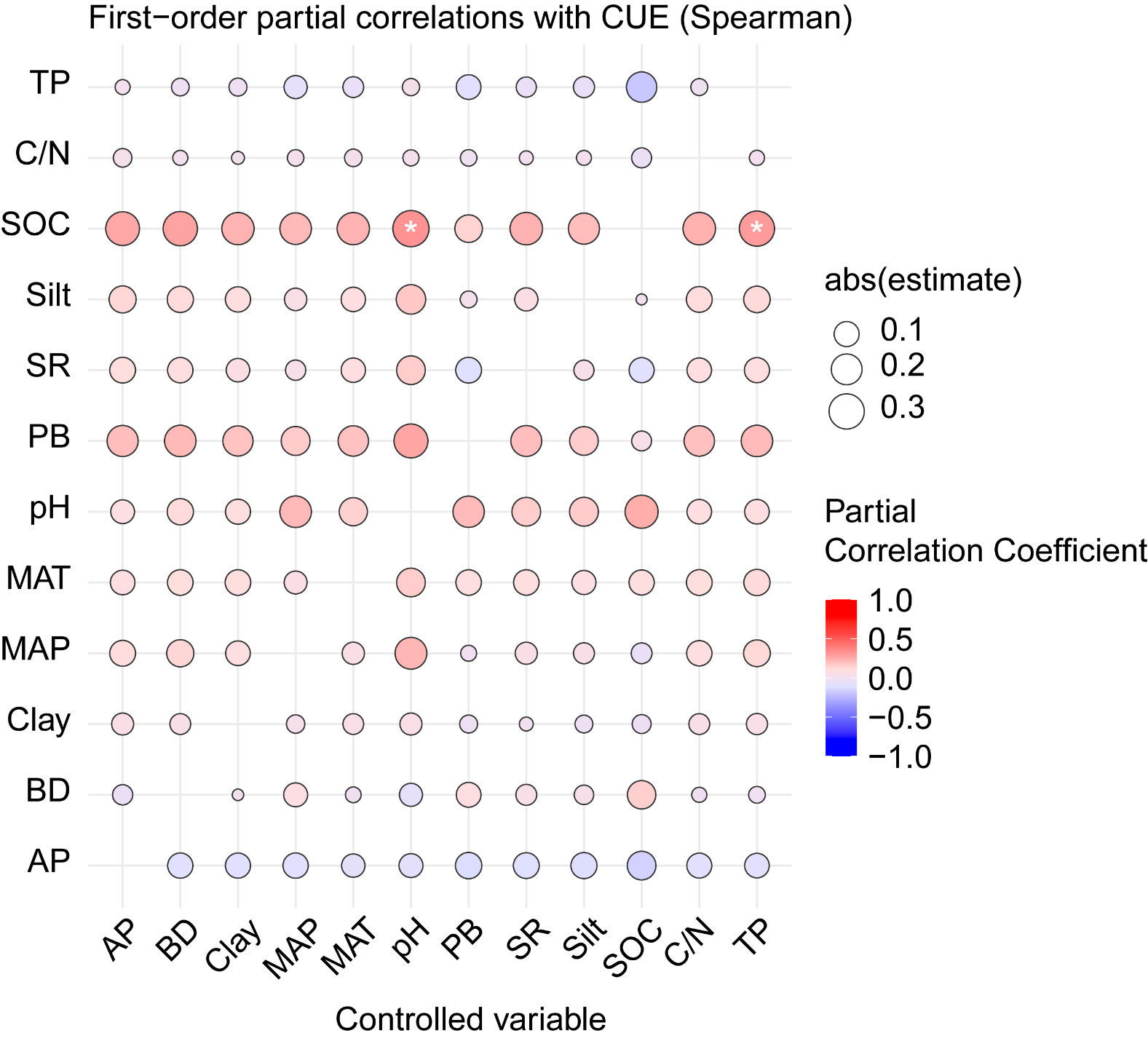
**

**Supplementary Figure 1**. Heatmap of first-order partial correlation coefficients (ρ) between environmental variables and microbial CUE. Values represent partial correlations estimated while controlling for all other variables. Significance testing was performed using the Benjamini–Hochberg method for FDR-adjusted; * *p* < 0.05; ** *p* < 0.01; *** *p* < 0.001. Warm colors denote positive correlations and cool colors denote negative correlations. All variables were Z-scored, and factors with strong collinearity (VIF > 10) were removed (TN, C/P, and AI). SOC, soil organic carbon; TN, soil total nitrogen; TP, soil total phosphorus; AP, soil available phosphorus; C/N, soil organic carbon to total nitrogen; C/P, soil organic carbon to total phosphorus; MAP, mean annual precipitation; MAT, mean annual temperature; AI, aridity index; BD, bulk density; PB, plant biomass; SR, plant richness.


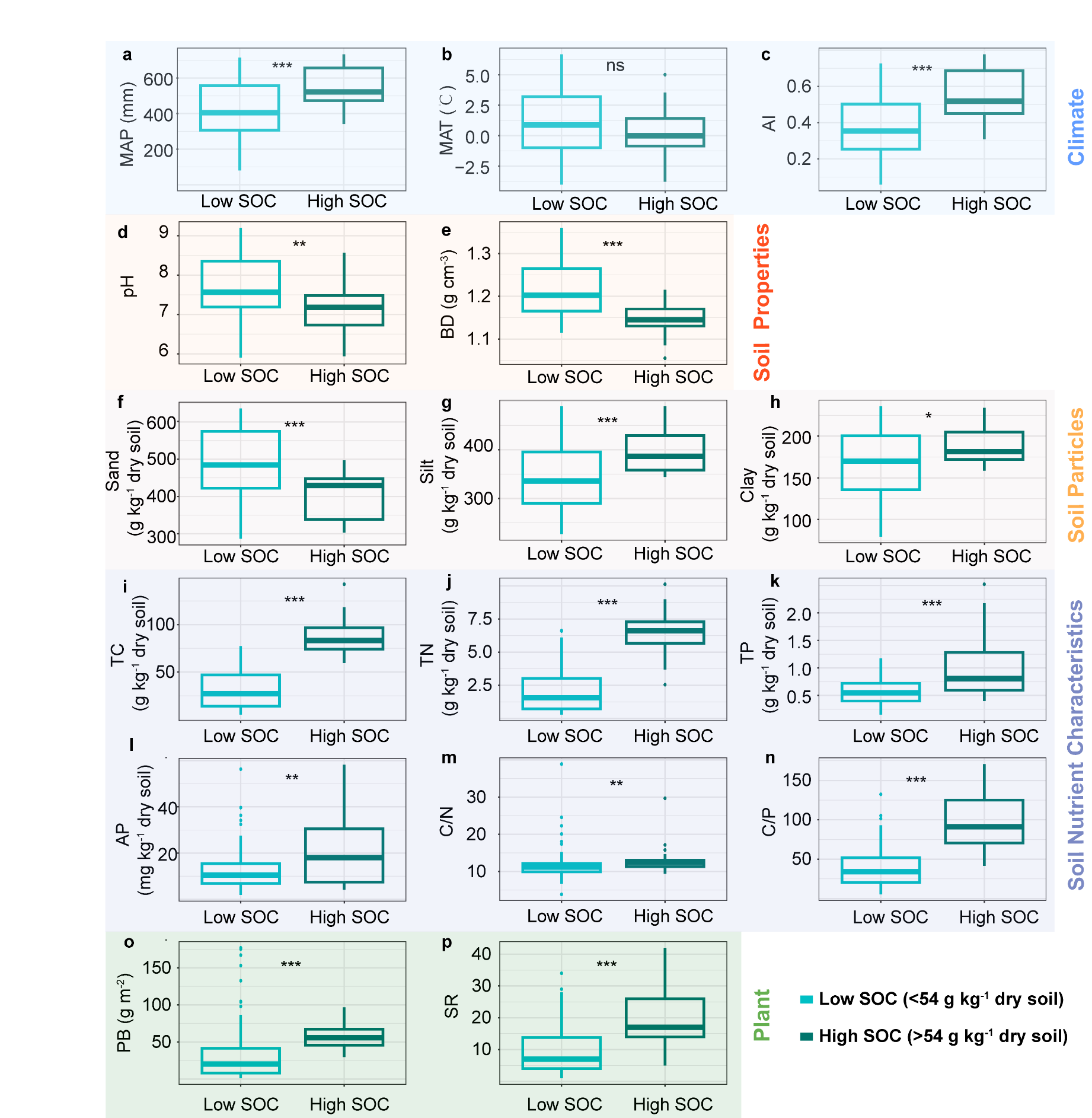


**Supplementary Figure 2**. Differences in environmental factors between low SOC and high SOC soils. Boxplots show median, interquartile range and outliers; significance was assessed using Mann–Whitney U test; * *p* < 0.05; ** *p* < 0.01; *** *p* < 0.001. MAP, mean annual precipitation; MAT, mean annual temperature; AI, aridity index; BD, bulk density; TC, soil total carbon; SOC, soil organic carbon; TN, soil total nitrogen; TP, soil total phosphorus; AP, soil available phosphorus; C/N, soil organic carbon to total nitrogen; C/P, soil organic carbon to total phosphorus; PB, plant biomass; SR, plant richness.


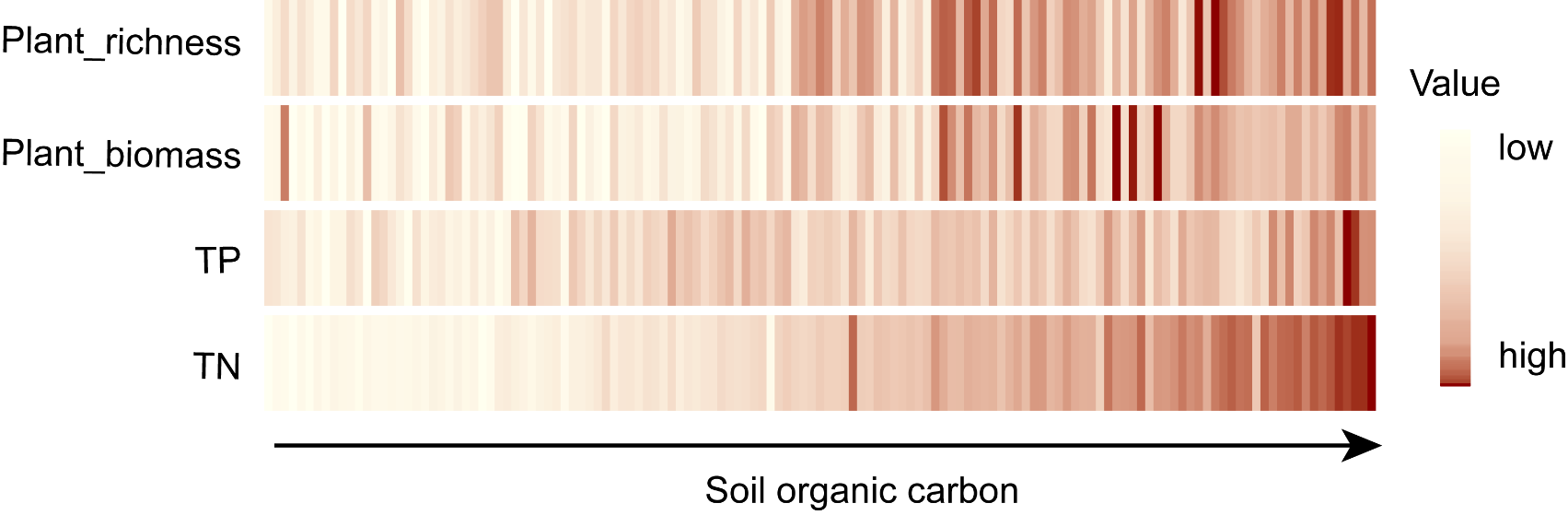


**Supplementary Figure 3**. Changes in plant biomass, plant richness, soil total nitrogen, and total phosphorus along the SOC gradient. SOC, soil organic carbon; TN, soil total nitrogen; TP, soil total phosphorus.

**
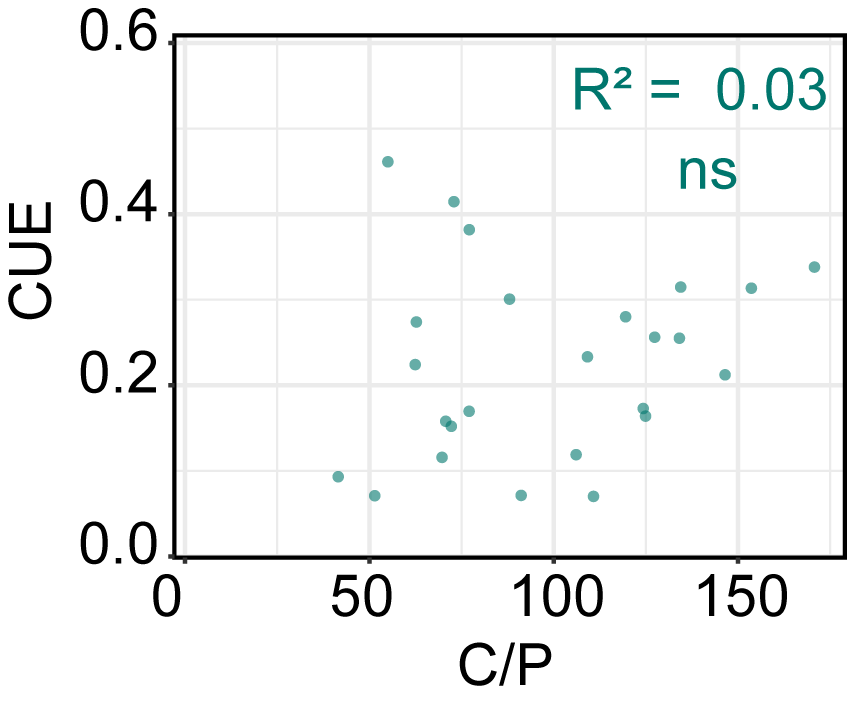
**

**Supplementary Figure 4**. In high organic carbon soils, the relationship between the soil C/P ratio and microbial CUE, based on frequency theory. CUE, carbon use efficiency; C/P, soil organic carbon to total phosphorus. ns, *p* > 0.05.

**
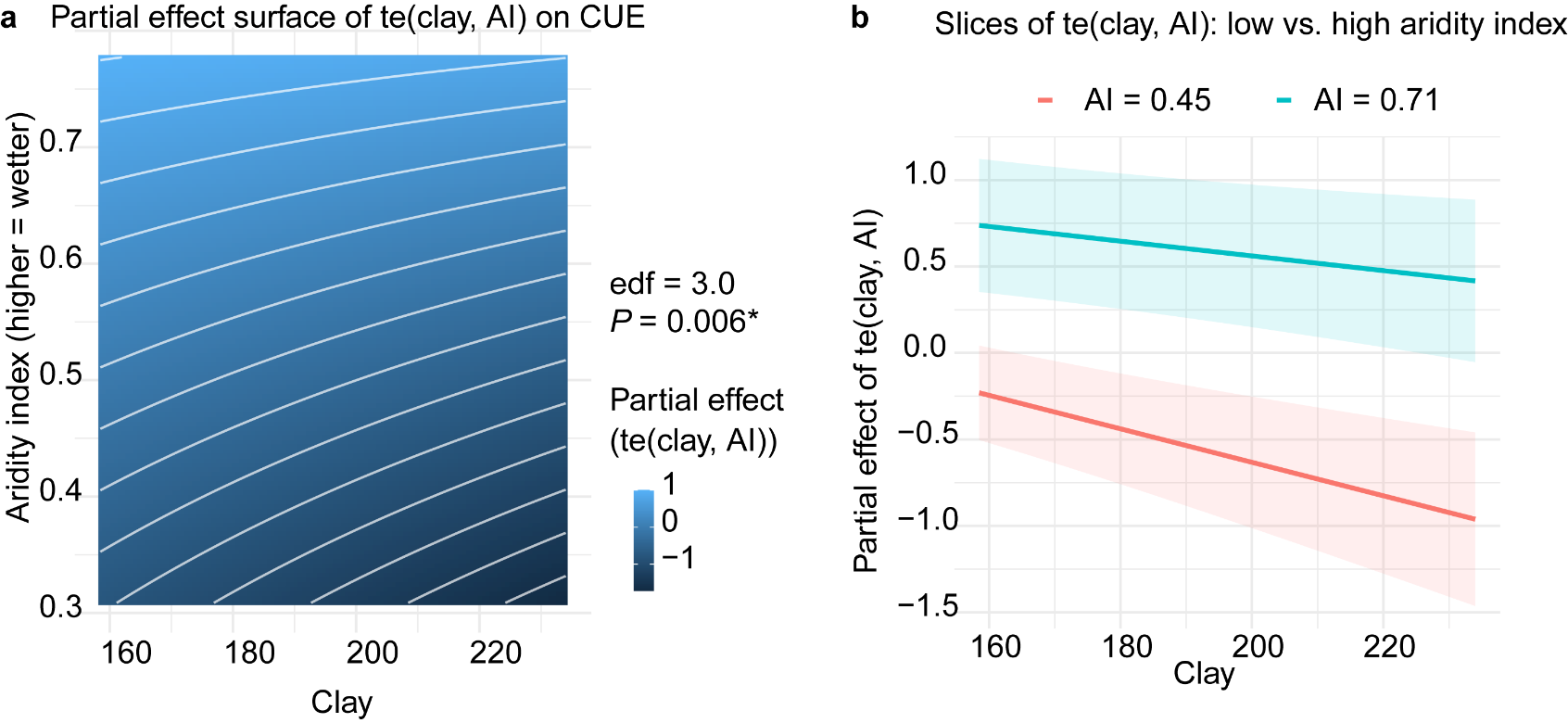
**

**Supplementary Figure 5**. a) Partial effect surface of te (clay, AI) on microbial CUE. Colors/contours show the partial effect of the bivariate smooth with other covariates held at their means (edf = 3.0, F = 7.58, *p* = 0.0062). b) Slices of te (clay, AI) at low/high AI. Shaded ribbons denote approximate 95% CIs; other covariates fixed at their means. AI (aridity index) is defined as the ratio of precipitation to potential evapotranspiration. A higher aridity index indicates wetter conditions.


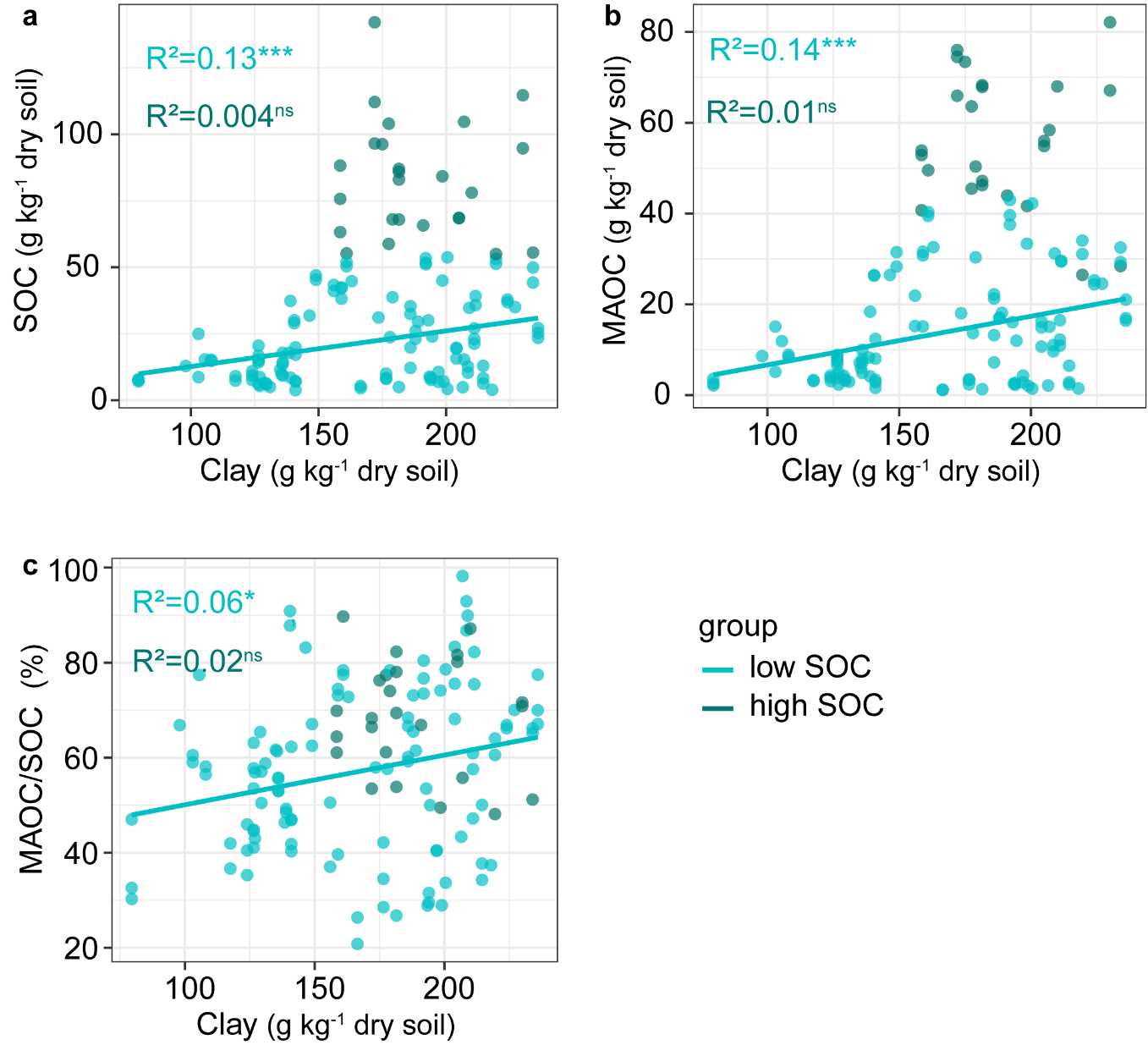


**Supplementary Figure 6**. The relationships between SOC, MAOC, SOC/MAOC and clay contents in low SOC and high SOC soils. Solid lines indicate significant linear fitting, and no lines indicate non-significant fitting; ns *p* > 0.05; * *p* < 0.05; ** *p* < 0.01; *** *p* < 0.001. SOC, soil organic carbon; MAOC, mineral-associated organic carbon. Low SOC (SOC < 54 g C kg ^-1^ dry soil); High SOC (SOC > 54 g C kg ^-1^ dry soil).

**
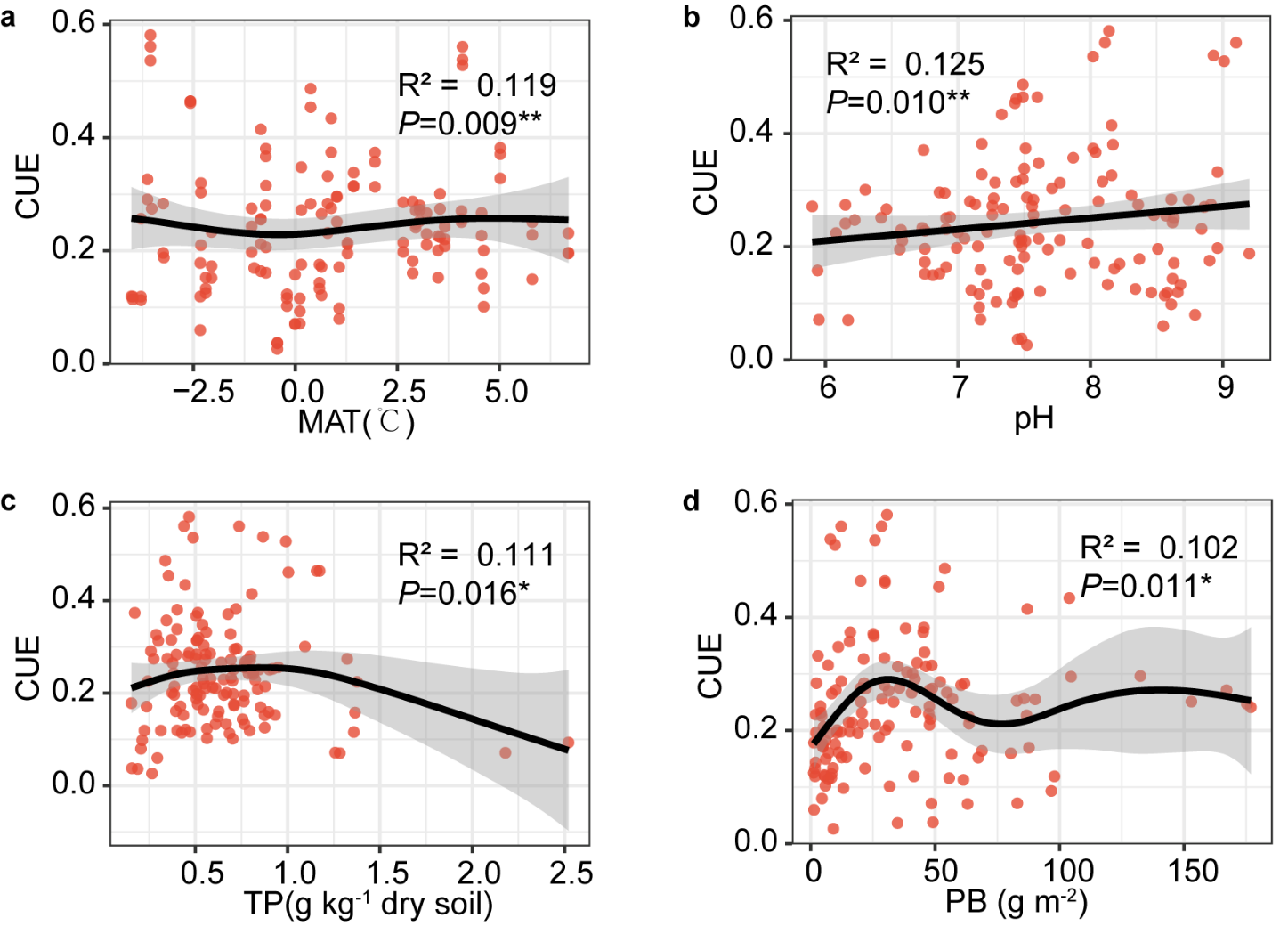
**

**Supplementary Figure 7**. Relationships between soil microbial CUE and environmental factors. a) Mean annual temperature, b) Soil pH, c) Soil total phosphorus, and d) Plant biomass. Black lines indicate nonlinear trends fitted by a generalized additive model (GAM), with shaded areas showing confidence intervals. * *p* < 0.05; ** *p* < 0.01; *** *p* < 0.001. For each variable, we fitted three models: linear, quadratic, and Generalized Additive Model (GAM). The optimal model was selected based on the Akaike Information Criterion (AIC); the model with the lowest AIC value was chosen. If the difference in AIC between the best and second-best model was less than 2, the simpler model was selected. Full statistics are provided in Supplementary Table 5.

**Supplementary Table 1**. Description of soil microbial CUE and factor values across all samples on the Qinghai–Tibetan Plateau.

| Variable | Minimum | Maximum | Range | Mean |
| --- | --- | --- | --- | --- |
| Soil Microbial CUE | 0.03 | 0.58 | 0.55 | 0.24 |
| Longitude (°E) | 79.82 | 102.59 | 22.77 | 94.13 |
| Latitude (°N) | 28.32 | 37.64 | 9.32 | 32.54 |
| Elevation (m) | 2505.00 | 4961.00 | 2456.00 | 4032.37 |
| MAT (ºC) | -4.02 | 6.69 | 10.71 | 0.8 |
| MAP (mm year^-1^) | 81.00 | 733.00 | 652.00 | 449.79 |
| Aridity index | 0.06 | 0.78 | 0.72 | 0.40 |
| Soil pH | 5.90 | 9.20 | 3.3 | 7.60 |
| Bulk density (g cm^-3^) | 1.06 | 1.36 | 0.30 | 1.20 |
| Soil sand content (g kg^-1^) | 286.50 | 636.00 | 349.50 | 474.76 |
| Soil clay content (g kg^-1^) | 79.50 | 236.00 | 156.50 | 171.38 |
| Soil silt content (g kg^-1^) | 227.00 | 489.50 | 262.50 | 354.60 |
| SOC (g kg^-1^) | 3.84 | 142.12 | 138.28 | 32.98 |
| TC (g kg^-1^) | 5.28 | 142.49 | 137.21 | 41.10 |
| TN (g kg^-1^) | 0.30 | 10.12 | 9.82 | 2.84 |
| TP (g kg^-1^) | 0.15 | 2.50 | 2.35 | 0.64 |
| AP (mg kg^-1^) | 1.86 | 58.23 | 56.37 | 14.19 |

CUE, Carbon Use Efficiency; MAT, Mean Annual Temperature; MAP, Mean Annual Precipitation; SOC, Soil Organic Carbon; TC, Soil Total Carbon; TN, Soil Total Nitrogen; TP, Soil Total Phosphorus; AP, Soil Available Phosphorus.

**Supplementary Table 2**. Best threshold models for soil organic carbon.

| Threshold Model | AIC | Threshold | Selection Threshold | | <= Threshold | | > Threshold | |
| --- | --- | --- | --- | --- | --- | --- | --- | --- |
|  |  |  |  |  | R^2^ | *p* | R^2^ | *p* |
| segmented | -257.18 | 49.91 | 54.355 | | 0.24 | *** | 0.49 | *** |
| stegmented | -257.24 | 58.80 |  | |  |  |  |  |
| step | -231.18 | 19.73 |  |  | |  |  |  |

Variables with their corresponding AIC values after threshold models are shown. Lower AIC values indicate a better fit of the model. A difference in AIC values of less than 2 between the two models suggests no substantial difference; therefore, the final threshold for soil organic carbon was determined as the mean of the thresholds from both models. The R² and *p* represent the goodness of fit and significance of linear regressions on both sides of the threshold. **p* < 0.05; ***p* < 0.01; ****p* < 0.001.

**Supplementary Table 3**. Effect sizes in the piecewise structural equation modeling (piecewise SEM) for low-SOC soils.

| Predictor | Effect type | Effect size | SE_boot |
| --- | --- | --- | --- |
| MAP | direct | 0.19 | 0.11 |
|  | indirect | -0.05 | 0.09 |
|  | total | 0.14 | 0.07 |
| MAT | direct | 0.00 | 0.00 |
|  | indirect | -0.10 | 0.05 |
|  | total | -0.10 | 0.05 |
| pH | direct | 0.43 | 0.13 |
|  | indirect | -0.08 | 0.03 |
|  | total | 0.36 | 0.14 |
| Clay | direct | 0.00 | 0.00 |
|  | indirect | 0.13 | 0.04 |
|  | total | 0.13 | 0.04 |
| TP | direct | 0.26 | 0.10 |
|  | indirect | -0.09 | 0.04 |
|  | total | 0.16 | 0.12 |
| C/P | direct | 0.53 | 0.10 |
|  | indirect | 0.00 | 0.00 |
|  | total | 0.53 | 0.10 |
| Plant biomass | direct | 0.00 | 0.00 |
|  | indirect | 0.24 | 0.07 |
|  | total | 0.24 | 0.07 |

SE_boot represents bootstrap standard error, which was estimated using a non-parametric bootstrap approach with 5,000 iterations. MAT, Mean Annual Temperature; MAP, Mean Annual Precipitation; TP, Soil Total Phosphorus; C/P, Soil Organic Carbon to Total Phosphorus.

**Supplementary Table 4**. Effect sizes in the piecewise structural equation modeling (piecewise SEM) for high-SOC soils.

| Predictor | Effect type | Effect size | SE_boot |
| --- | --- | --- | --- |
| MAP | direct | 0.00 | 0.00 |
|  | indirect | -0.30 | 0.10 |
|  | total | -0.30 | 0.10 |
| MAT | direct | 0.34 | 0.15 |
|  | indirect | 0.00 | 0.00 |
|  | total | 0.34 | 0.15 |
| Clay | direct | 0.00 | 0.00 |
|  | indirect | -0.04 | 0.10 |
|  | total | -0.04 | 0.10 |
| AP | direct | 0.00 | 0.00 |
|  | indirect | -0.01 | 0.05 |
|  | total | -0.01 | 0.05 |
| TP | direct | -0.94 | 0.34 |
|  | indirect | 0.38 | 0.31 |
|  | total | -0.56 | 0.14 |
| C/P | direct | -0.50 | 0.35 |
|  | indirect | 0.00 | 0.00 |
|  | total | -0.50 | 0.35 |
| Plant biomass | direct | 0.00 | 0.00 |
|  | indirect | -0.02 | 0.12 |
|  | total | -0.02 | 0.12 |

SE_boot represents bootstrap standard error, which was estimated using a non-parametric bootstrap approach with 5,000 iterations. MAT, Mean Annual Temperature; MAP, Mean Annual Precipitation; TP, Soil Total Phosphorus; C/P, Soil Organic Carbon to Total Phosphorus.

**Supplementary Table 5**. Best fitting models for each variable and microbial CUE.

| Variable | Linear AIC | Quadratic AIC | GAM AIC | Model Selection |
| --- | --- | --- | --- | --- |
| SOC | -214.987 | -250.726 | -254.555 | GAM |
| pH | -194.580 | -192.818 | -205.544 | GAM |
| MAT | -192.292 | -191.082 | -202.955 | GAM |
| TP | -192.659 | -196.313 | -203.503 | GAM |
| Plant biomass | -192.720 | -192.320 | -204.293 | GAM |
| TN | -193.034 | -218.673 | -217.663 | Quadratic |
| MAP | -193.300 | -197.639 | -206.580 | GAM |
| AI | -192.921 | -200.981 | -215.090 | GAM |
| Silt | -192.567 | -190.568 | -192.890 | Linear |
| Clay | -192.158 | -190.605 | -198.502 | GAM |
| BD | -192.257 | -190.258 | -220.784 | GAM |

Variables with their corresponding AIC values after fitting linear and nonlinear (Quadratic and GAM) models are shown. Lower AIC values indicate a better fit of the model. A difference in AIC values of less than 2 between the two models suggests no substantial difference, preferring the selection of the simpler linear model. GAM, generalized additive model. MAT, Mean Annual Temperature; MAP, Mean Annual Precipitation; SOC, Soil Organic Carbon; TN, Soil Total Nitrogen; TP, Soil Total Phosphorus; AP, Soil Available Phosphorus; AI, Aridity Index; BD, Bulk Density.
